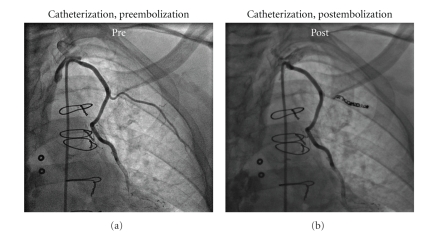# Impaired Myocardial Perfusion from Persistent Mammary Side Branches: A Role for Functional Imaging and Embolization

**DOI:** 10.4061/2010/203459

**Published:** 2010-09-13

**Authors:** Michael S. Firstenberg, Gregory Guy, Charles Bush, Subha V. Raman

**Affiliations:** ^1^Division of Cardiothoracic Surgery, The Ohio State University Medical Center, N817 Doan Hall, Columbus, OH 43210, USA; ^2^Department of Radiology, The Ohio State University Medical Center, N817 Doan Hall, Columbus, OH 43210, USA; ^3^Department of Cardiology, The Ohio State University Medical Center, N817 Doan Hall, Columbus, OH 43210, USA

## Abstract

The diagnosis and management of ischemic symptoms in patients after coronary artery bypass surgery can be challenging. It has been hypothesized that persistent branches of the internal mammary artery can divert flow from the left anterior descending artery and cause symptoms. We present a case in which successful coil embolization of a side branch improved flow and clinical symptoms. Side branch embolization might be a useful treatment option and should be considered in the management of symptomatic patients with a patent mammary graft.

## 1. Introduction

The evaluation and management of potentially ischemic symptoms in patients who have undergone previous coronary artery bypass surgery can be challenging. While progression of native disease, graft failure, or incomplete revascularization is often factors, unusual problems can cause recurrence of symptoms. The use of the internal mammary artery is the most durable and reliable conduit for left anterior descending revascularization, nevertheless, this technique is not without long-term complications. As the IMA is harvested, intercostal branches are interrupted however, persistent branches, typically the first intercostal, have been associated with IMA dysfunction—although proof of cause and effect is debatable. We present a case with functional imaging that supports a pathophysiologic relationship.

## 2. Case

Our patient is a 69-year-old gentleman who underwent previous CABG over 10 years prior and included a LIMA-LAD. He presented with chest discomfort and poor exercise tolerance despite maximal medical therapy that had been getting worse over the preceding 6 months. As part of a work-up, an MRI viability study was performed which showed inducible ischemia in the anterior wall distribution. Cardiac catheterization showed a patent LIMA-LAD and a persistent 1st intercostal branch (Figure 1(a)). Percutaneous coil embolization was performed (Figure 1(b)). Immediately postprocedure he had minor and transient (<24 hours) chest discomfort. This was attributed to the embolization of the intercostal as his cardiac enzymes were negative and his ECG was unchanged. He was subsequently discharged. At a 1-month followup visit, the patient reported reduced angina and increased functional capacity, with corresponding diffuse global, nonspecific, improvement in endocardial perfusion.

## 3. Discussion

Despite it established durability as an effective means of revascularization, the use of the LIMA-LAD is not without problems. Even though graft failure is rare, recurrence of symptoms can occur even in the face of a patent conduit. One potential cause of recurrent symptoms can be an anomalous or persistent, chest wall branch causing flow “steal.” The relationship between ischemic symptoms and these branches is debatable both clinically [1] and experimentally [2, 3] based upon flow reserve studies. The physiologic significance of these branches has also been questioned in the sense that many patients have these branches, but few have problems from them [4]. Nevertheless, there have been reports of clinical improvements following direct ligation [5] or coil embolization [6].

Our case demonstrates impaired anterior wall myocardial perfusion in the setting of a patent LIMA-LAD and an anomalous LIMA side branch. Even though symptom improvement is subjective, we demonstrated, with advanced MRI imaging, improvement in perfusion following embolization of the branch. These findings support the hypothesis that these branches cannot only compromise myocardial perfusion, but also their interruption can improve perfusion. Correlating the clinical significance to the imaging findings is debatable, but it should be intuitive that in patients with marginal myocardial blood flow from severe and diffuse disease would benefit from any and all addition perfusion. Unfortunately, a limitation in this case is the lack of a clearly defined territory of improved perfusion, but rather a subjective and diffuse impression that perfusion was improved globally after ligation of the side branch. This concept supports the hypothesis that this patient's symptoms, despite a patent IMA graft many years after his initial surgery, progressed at a microcirculatory level to the point in which he finally became symptomatic. Hence, whatever additional blood flow was accomplished by acutely eliminating the side branch shunt was, as we suspect, sufficient to overcome his angina. Nevertheless, there still is controversy in the area that needs to be explored [7].

## 4. Conclusions

Patients with recurrent symptoms after CABG who have a patent LIMA-LAD should have functional studies to determine potential ischemic territories. If LIMA side branches are found, ligation or embolization should be considered as a means of improving myocardial flow. In patients with diffuse and severe disease in which any improvement in flow may alleviate symptoms, this intervention might provide a clinical benefit. Hopefully, this case will spark further studies in this area using advanced quantitative perfusion-based imaging such as CMR.

## Figures and Tables

**Figure 1:**